# PasoDoble, a Proposed Dance/Music for People With Parkinson's Disease and Their Caregivers

**DOI:** 10.3389/fneur.2020.567891

**Published:** 2020-11-12

**Authors:** Lydia Giménez-Llort, Lidia Castillo-Mariqueo

**Affiliations:** ^1^Department of Psychiatry and Forensic Medicine, School of Medicine, Universitat Autònoma de Barcelona, Barcelona, Spain; ^2^Institut de Neurociències, Universitat Autònoma de Barcelona, Barcelona, Spain

**Keywords:** Parkisnon's disease, prevention, rehabilitation, quality of life, caregiver, dance and movement psychotherapy, family-centered care, home care

## Abstract

Managing the heterogeneity of Parkinson's disease symptoms and its progressive nature demands strategies targeting the hallmark disrupted neurotransmission but also the comorbid derangements and bolstering neuroprotection and regeneration. Strong efforts are done to find disease-modifying strategies, since slowing disease progression is not enough to hamper its burden and some motor symptoms are resistant to dopamine-replacement therapy. The inclusion of non-pharmacological strategies can provide such a multitarget umbrella approach. The silent long-term biological process that precedes the clinical onset of disease is a challenge but also an opportunity to reinforce healthy lifestyle known to exert preventive/therapeutic effects. These non-pharmacological strategies are foreseen as able to reduce the prevalence and the global impact of long-term diseases demanding strong management of patient-caregiver quality of life. In this regard, European guidelines for Parkinson's disease recommend physical-related activities such as aerobic exercise and dancing known to improve functional mobility and balance in patients. Here, we propose “PasoDoble,” a novel dance/music patient-caregiver intervention with additional preventive value. The rationale is founded on evidence-based therapeutic benefits of dance/music therapy and the singular features of this widely extended Hispanic dance/music targeting motor symptoms, mood/cognition, and socialization: (i) As a dance, an easy and simple double-step pattern (back-and-forward and lateral movements) that evolves from a spontaneous individual dance to a partnered dancing, performed in social groups and involving dancing-figures of increasing complexity; (ii) “PasoDoble,” as a music that can be sung, has musical rhythmicity with high groove and familiarity that will help to synchronize the steps to the rhythm of music; (iii) Widely extended (Spain, Mexico, Puerto Rico, Colombia, and USA) and easy-to-learn for others. As a regular dancing “PasoDoble” can improve and preserve function, mood and socialization, as an intervention the method is structured to improve gait and balance; facilitate movement, reaching and grasping; muscle power and joint mobility; reduce of risk of falls, and increase of aerobic capacity. Finally, this easy-to-implement into patient care and free-living environments (elderly social centers, home care) rehabilitation programs can promote positive emotions and self-esteem, with added general improvement of social attachment and recognition, thus improving the quality of life of patient-caregiver.

## Introduction

Parkinson's disease (PD), as most aging associated neurodegenerative pathologies, has a heterogeneous presentation. Besides the hallmark motor symptoms, sensorial, cognitive, and psychiatric alterations can also be developed (1, 2). Managing such a diversity of symptoms and progressive nature demands strategies targeting the hallmark disrupted neurotransmission but also the comorbid derangements and the bolstering of neuroprotection and regeneration (3). Currently, there are various treatment strategies to address the symptoms and progression of PD but pharmacotherapy is the treatment of choice (2–4).

The dopaminergic therapy targets the prominent loss of dopaminergic neurons in the substantia nigra that caused a deficiency of dopamine and results in the main clinical symptoms that characterize the disease: tremor, bradykinesia, rigidity, and postural instability (4–6). Besides, other typical motor symptoms can be observed, such as an altered gait pattern, freezing of walking steps and deficits in coordination, directly impacting general motor control, and mobility of the entire body of the individuals who suffer from it (7). Thus, these motor disorders are dramatically evidenced in the gait of the individual as the disease progresses, recognizing an “akinetic-rigid gait” when these alterations are accentuated (8). Gait disturbances include decreased steps speed with decreased length as well, a narrow support base, and a hunched posture that affects the neck, shoulders, and trunk. Also, the oscillation of the arms and the rhythmic movement of the upper and lower cingulum are reduced, causing the arms to remain in an adducted position and flexed toward the trunk. In the lower extremities, the feet are raised less than normal, which can lead to walking intermittently causing greater variability in each step and therefore the gait time spent increases (4, 7, 8). Freezing usually occurs at the start of the gait but can also appear when turning or approaching an obstacle. It develops from early stages and usually decreases with medication in less severe stages. While the adaptation mechanisms and postural adjustments along with the dynamic balance are altered during the execution of the steps in the gait (6).

With regards to non-motor symptoms (NMS) of PD, growing number of literature (3, 9) consistently describes REM sleep behavior disorder and constipation; mood and affect disorders with anxiety and depression; sensory disfunction with hyposmia and taste loss, as early non-motor features preceding onset of motor symptoms of the disease (10). Excessive daytime sleepiness, fatigue, pain, apathy, and dysfunction of other autonomic responses such as urinary dysfunction and orthostatic hypotension, excessive sweating, are also commonly reported in the early-stage PD. Neuroimaging of NMS in PD showing early affectation of monoamine and cortical systems beyond the nigrostriatal dopaminergic pathway supports these broad spectrum of symptoms that during the progress of the disease will also involve cognitive domains, with executive dysfunctions (11), deficits in procedural learning (12, 13), visual hallucinations and dementia, as well as complex behavioral disorders (14). Executive functions and attention seem to be associated with gait and balance, a relationship that is conspicuous in normal aging and in neurodegenerative diseases that affect cognition and/or motor functions (15). However, other reports found no correlation between NMS burden and motor severity, age, or gender (10).

At the therapeutical level, strong efforts are done in order to find disease-modifying strategies, since slowing disease progression is not enough to hamper the burden of disease and some symptoms are resistant to dopamine-replacement therapy (1–4, 16). The above mentioned prodromal stages unveiled by NMS point at new scenarios for early intervention, but inability to make a conclusive diagnosis at the earliest stages as well as difficulties in the management of symptoms at advanced stages of the disease are still important clinical challenges (1). In this sense, the inclusion of non-pharmacological strategies and managing PD with a multidisciplinary perspective can contribute to provide such a multitarget umbrella approach. Thus, the silent long-term biological process that precedes the clinical onset of disease is a challenge but also an opportunity to reinforce healthy lifestyle known to exert preventive/therapeutic effects. For its part, non-pharmacological treatment requires the intervention of several disciplines, among which is therapeutic exercise, use of auditory, visual and somatosensorial stimuli, care strategies and complementary therapies, which reflects the wide range of options and alternatives of choice. These non-pharmacological strategies are foreseen as able to reduce the prevalence and the global impact of long-term diseases demanding strong management of patient-caregiver quality of life. In this regard, European guidelines for Parkinson's disease recommend physical-related activities such as aerobic exercise and dancing known to improve functional mobility and balance in patients (17).

## “Pasodoble”, A Proposed Dance/Music Intervention for People With Parkinson'S Disease and Their Caregivers

PD rehabilitation programs for people with PD include combined multimodal exercises of strength, balance, coordination, and/or aerobic training for the improvement of physical and motor condition (18, 19). It is possible to create circuits with a great variety of exercises, for example, those used in walking and overcoming obstacles that can provide benefits in patients with mild and moderate PD, reducing intrinsic fragility and providing greater possibilities for the control of dynamic balance in the performance of the steps in each stride (20, 21).

In this context, pasodoble is a type of ballroom dance that is characterized by its base step consisting of walking, so integrates many features of therapeutic exercise. In this traditional dance, the couple is of great importance since figures of eight are made in eight steps in a coordinated and harmonious way to the rhythm of the music (2 × 4). The important thing is to perform the dance steps with the upright posture, raising the chest and contracting the abdomen for proper control of the trunk. Some of the basic figures that can be performed include walking back and forth on the same site, making turns, and incorporating lateral movements. Then it progresses to separation and return of the couple, walk of the woman and crossed steps at different times. Finally, fast turns are made with forward, backward, and lateral movements in 8 times.

**Figure 1:**
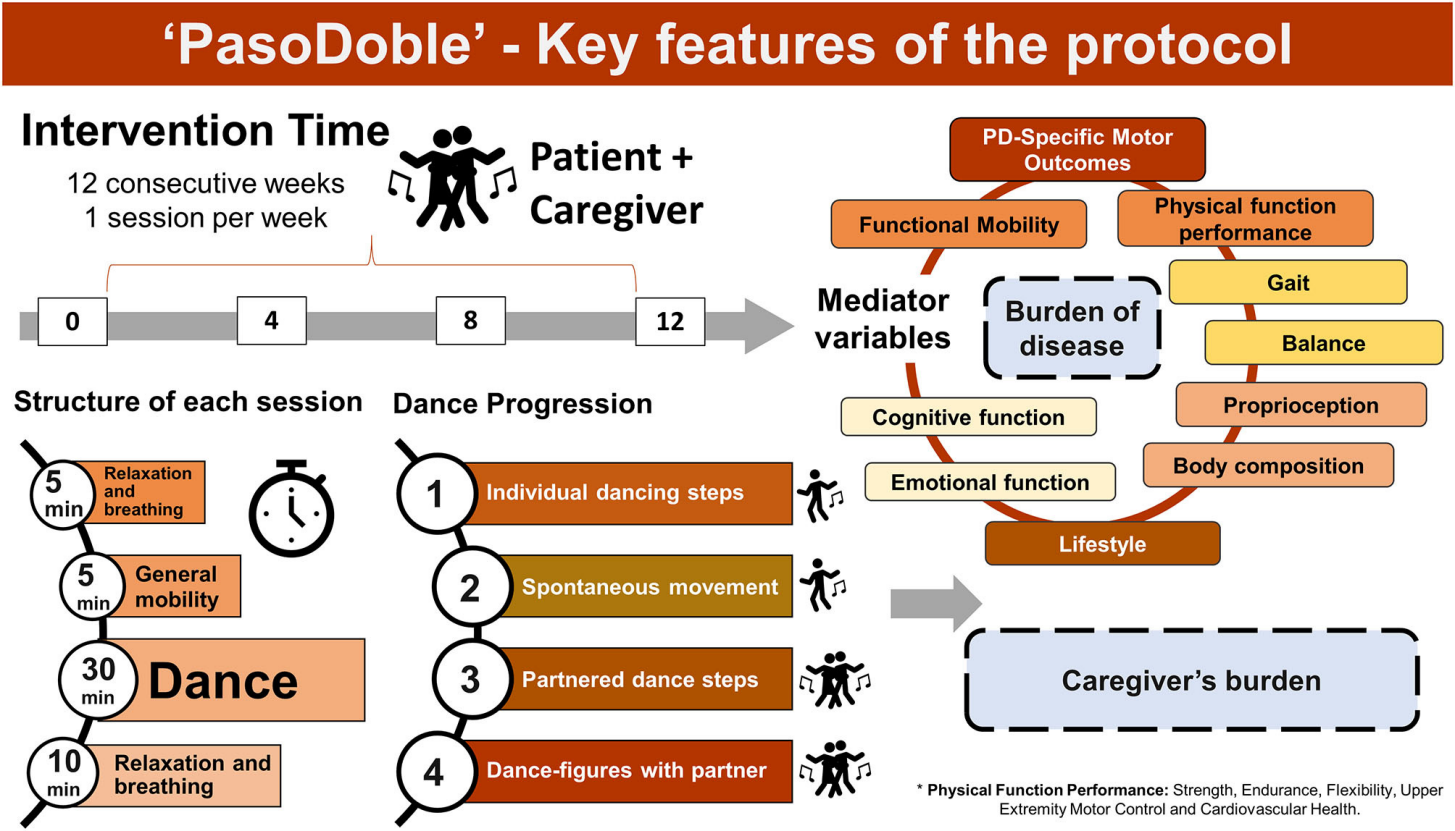
Key features of the ‘PasoDoble' protocol.

**Figure 2:**
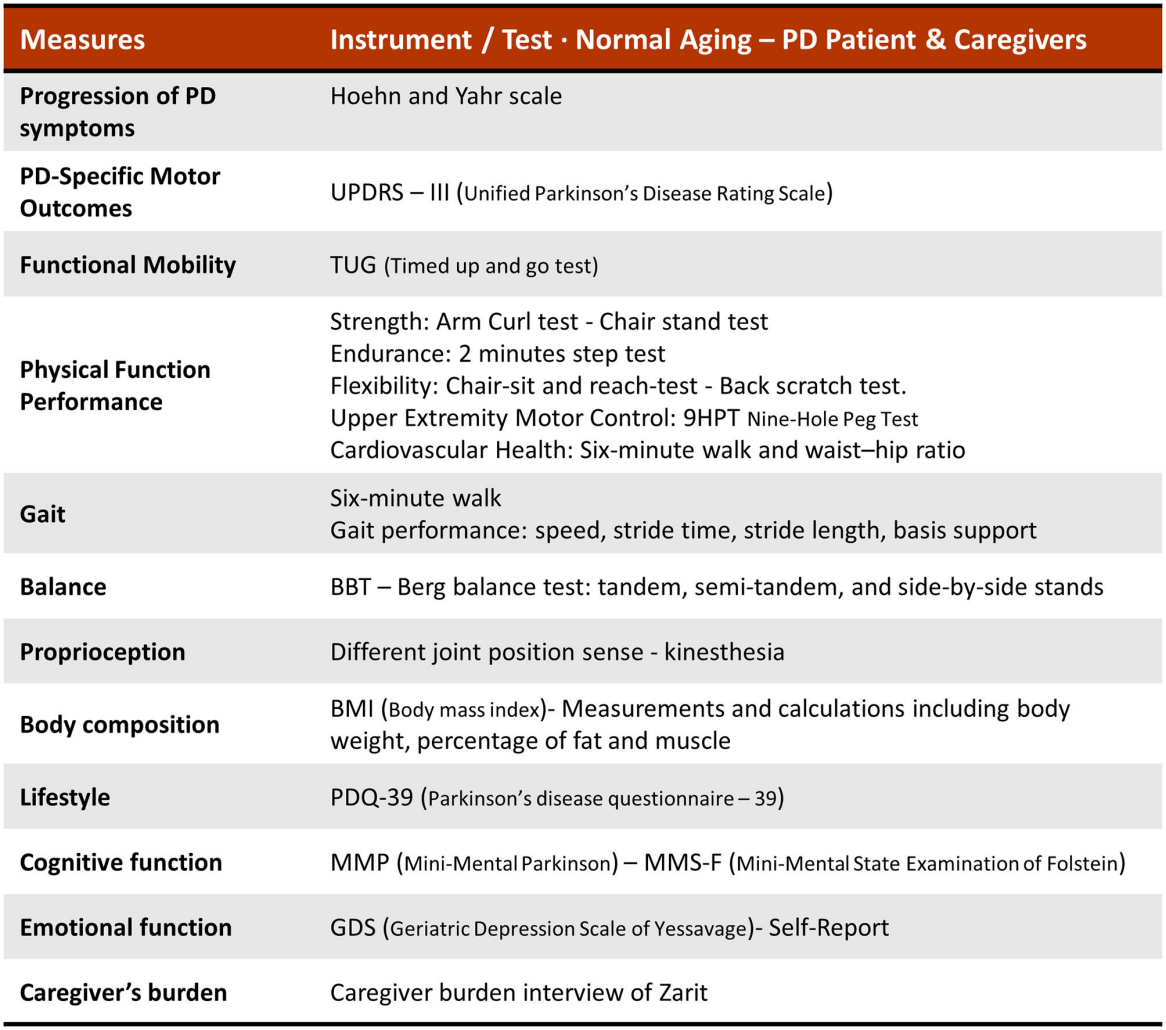
Measures and tools to evaluate the effects of ‘PasoDoble' in normal aging, the PD patient and the caregivers.

Based on this traditional pasodoble dance/music, here, we propose “PasoDoble,” a novel dance/music patient-caregiver intervention with additional preventive value. We also want to highly its potential value for maintenance of physical well-being as a regular dancing in the lifestyle of the Hispanic elderly. We aim to provide a methodological scenario (see Figure 1) and the theoretical fundaments (see Box 1) for the benefits of this dance and method (see Box 2) improving gait, balance, and functional disturbances in people with Parkinson's disease but also patient-caregiver quality of life, as a family caregiving intervention. However, the investigation of this approach as an intervention targeting PD is still a work that is currently in progress (see Figure 2 for Measures and Tools).

Box 1 **Scientific rationale of “PasoDoble.”**

**The evidence-based therapeutical benefits of dance/movement therapy and the singularity of PasoDoble features**. The European physiotherapy guideline for Parkinson's disease recommends dance as “a meaningful approach to improve functional mobility and balance” (17). According to the stage of progression of the disease, the proposed **time-line of intervention** relays on four **key areas**:

1) Cueing strategies to improve gait,

2) Cognitive movement strategies to improve transfer,

3) Exercises to improve balance and

4) Training of joint mobility and muscle power to improve physical capacity (22).

In fact, since the early work by Westbrook and McKibben (23), **dance/movement therapy** has been reported been more effective than exercise in the outpatient treatment of patients with Parkinson's disease. Underlying **neural mechanisms** are a research area of growing interest (24) and it has been speculated that regular dancing practice may facilitate activation of sensory-motor areas impaired in the brain of people with PD (25). On parallel activation by means of **music** of brain areas such as amygdala, nucleus accumbens, hypothalamus, hippocampus, insula, cingulate cortex, and orbitofrontal cortex are pointed out as those providing emotional benefits (26). Besides, in **partnered dancing**, dance partner and music may exert additive effects on achievement of **rhythmicity** since they both provide **spatial external cues and timeframes** that may facilitate movement initiation and execution in a PD-brain with impaired basal-ganglia that leads not only to movement disturbances but also worse rhythm perception as compared to controls (27). Motor control, expertise and action-perception links, sensory cues for physical contact, synchronization of the steps to the rhythm of music, **familiarity** with the music, **groove** that compels the body to move, and motor learning processes are also considered among key factors in neurocognitive control of dance perception and performance (24, 28). Furthermore, partnered dancing as a **social dance practice** in **community settings** has been described as an effective and attractive form of entertaining for patients with PD due to the level of physical interaction and amusement between partners and the social group.

Box 2 **The 5 key fundaments of the “PasoDoble” Method The “Pasodoble” methods is based on the 5 key features, that can be summarized as follows:**

i) As a dance, an **easy and simple double-step pattern** (back-and-forward and lateral movements) that evolves from a **spontaneous individual dance** to a **partnered dancing**, performed in social groups and involving dancing-figures of increasing complexity; Based in a simple walking pattern (2 beats, 8-to-8 steps, 2 × 4 rhythm), easy to translate into a training program. Its basic performance is spontaneous and flexible, not predefined routine from start to end, but guided by the rhythm of the music, with the directionality of movement including the **facilitatory effect of the partner**.
ii) “PasoDoble,” as **a music that can be sung**, has musical rhythmicity with **high groove and familiarity** that compels the body to move, overcoming the difficulties and tiredness that the parkinson's patient encounters with each movement. It also helps to synchronize the steps to the rhythm of music with the extra-guidance of the dance partner (caregiver);
iii) **Widely extended** (Spain, Mexico, Puerto Rico, Colombia, and USA) and **easy-to-learn** for others.
iv) While **as a regular dancing** “PasoDoble” can improve and preserve function, mood and socialization, **as an intervention** the “PasoDoble” method is structured to improve gait and balance; facilitate movement, reaching and grasping; muscle power and joint mobility; reduce of risk of falls, and increase of aerobic capacity.
v) Finally, this **easy-to-implement into patient care and free-living environments** (elderly social centers, home care) **rehabilitation program** can promote positive emotions and self-esteem, with added general improvement of **social attachment and recognition**, thus improving the **quality of life of patient-caregiver**.

The rationale of our proposal “PasoDoble” as a PD intervention is founded on evidence-based therapeutic benefits of dance/music therapy that will be discussed below. Also, it is based in the singular features of this widely extended Hispanic popular dance/music targeting motor symptoms, mood/cognition and socialization (see Box 2).

Rehabilitation programs for PD should be “goal-based” (aimed at practicing and learning specific activities in core areas), but it is necessary to identify a number of practical variables (intensity, specificity, complexity), as well as adaptations related to the individual characteristics of the patients according to the degree of involvement and severity that the disease has caused with its clinical manifestations of motor disorder.

The implementation and realization of the protocol “PasoDoble” include the following methodological aspects: firstly, where the dance takes place, it must provide the participants first security. It must have the physical requirements that provide a spacious and illuminated space to develop each of the sessions. The environment can be adapted for video-recordings and train the professional team (neurologist, nurse, occupational therapist, psychologist, kinesiologist, and professional dancers). According to precedent data in other dance/movement therapies, 1 h session, twice a week, 12 weeks, pre-test (Evaluation 0) and follow-up evaluation at 3, 6, 9, and 12 months would be the proposed duration by which an individual must engage in the intervention to elicit a therapeutic benefit. Here, we have designed the intervention also considering how dancing is scheduled in social clubs elderly people. The protocol lasts 12 consecutive weeks (a seasonal trimester) with a frequency of therapy of once a week with a structure and duration of each session as follows (see Figure 1): duration of 50 min (a total duration of 1 h if considering get started/get over) with an opening with relaxation and breathing (5 min), general mobilization (5 min), PasoDoble dancing (30 min), and a closing with relaxation and breathing. The dance progression during the weeks considers 4 phases of intervention or stages of training. Initially, simple and less complex movements will be performed until reaching the dance performance in pairs (1. spontaneous movement rhythmicity and 2. sequence of dance steps), then 3. partnered dance and finally 4. dance-figures with partner. That is, an intervention program would fit with a trimester proposal, and 1 session per week. In the context of Hispanic population, but not restricted to them, a widely extended habit of elder people is to enroll to social clubs, when once a week (usually Sundays) life music is performed and elderly dance with their own partners or other elderly attending the social event. Family associations for PD also have their own spaces were rehabilitation programs are performed. Here, the intervention time was designed to be perfectly adapted to this natural timings and habitudes, but under a structured protocol where decisions have been made by the therapist, not the participants. An important question in the inclusion criteria to be part in the program is to do a functional evaluation (pre-test) to determine the stage of PD (I, II, and II of the Hoehn and Yahr scale) so that the person can be allocated to the corresponding level. Age of participants should not be a criteria. Control of drug therapy must be done. The inclusion of a regular partner in this partnered dance provides an internal control group, at least for most functional variables. However, since the proposed method is also aimed to improve quality of life of caregivers, enrolling as the partners in the dance, self-reliant older adults would be the best control group, for burden of disease in PD patient and caregiver's burden as the

partner. As in many other proposals, blind evaluators, and pilot studies in real settings, are also advised.

Although non-pharmacological therapy has gained great importance, there is still little consensus on how to measure the analysis of therapies, being a wide and interesting field of research to address (29–31). The current ongoing research using this PasoDoble protocol is focused on clinical measures of PD and caregiver's health, such as motor symptoms, mood/cognition, and socialization (UPDRS, BBS, TUG, 6MWT, FOG, DGI, Sit-to-stand test, Beck, Zarit QoL). The instruments and specific tests for each variable are as mentioned in illustration 2. The expected result in a pre- post- analysis and as compared to control standard physical activity, include: improvement of gait and balance, facilitation of movement through external cues and specific movements, better coordination and stability with the partner, reaching and grasping, muscle power and joint mobility, reduction of risk of falls, increase of aerobic capacity (see Figure 1). This rehabilitation program will be easy to implement into patient care but also in already socially established free-living environments (community setting such as elders social centers, home) improving patient-caregiver quality of life by means of promotion of positive emotions and self-esteem with additional general improvement of social attachment and recognition.

Finally, limitations of the proposal, most of them in common with other non-pharmacological strategies, must also be mentioned here. Studies in older adults have shown that exercise outcomes, such as improved self-efficacy, are more strongly predicted by the social cognitive factors associated with exercise. In fact, in spite of being a common shortcoming, PasoDoble has an advantage, as the naïve ballroom dance (pasodoble) is already popular in elderly social centers, and part of its strength to be implemented as a preventive or rehabilitation program (with a schedule or protocol) relies on this “familiarity” or “social” aspect. So, social cognitive factors are part of the virtues of this dance and looked for, with purpose, as an intervention. The small number of participants, lack of partner for the interventions or dancers unfamiliar with PD can be also foreseen as a critical point. However, this kind of dance is so popular but also intimate, that it can be implemented to the minimum of one patient and his/her therapist. Regarding the time schedule, a 3-month duration of the intervention may be too short to see strong effects upon participation, and commitment over a 10-week period may be difficult, in this and other programs. As mentioned before, the initial proposal is established in 12 weeks (3 months) as it is a formula that fits trimestral (seasonal) activity proposals and easier to be committed with. With regards to the severity of PD, it may vary within the sample. Thus, heterogeneity in the level of impairment requires a prior accurate evaluation of the motor impairment, could affected participation but could be solved with the establishment of different groups or levels. The last, but not least, randomized controlled trials are necessary to better understand the effects of programs of dance therapy for older adults with and without PD, and this also applied to this proposed method that is currently under study.

## “Pasodoble” As A Form of Therapeutic Exercise for PD and Protective Strategy for Caregivers

In the following paragraphs we will provide further discussion on the benefits that can be derived as a form of therapeutic exercise programs for rehabilitation in PD, thereafter those that are in common with other dances, and finally we'll present the scenario of PasoDoble in this 2020–2030 Decade of Healthy Aging where family-centered care models are among the most important needs pointed out by UN.

The main objective of therapeutic exercise and rehabilitation is to improve and maintain the quality of life of people with PD. This is done by helping to improve or increase mobility, balance, gait, coordination, and thus maintaining the functional autonomy of the patient for a longer time. Educating their environment, family, caregivers, and community will also contribute to finally promote an active role in the rehabilitation process (18, 32). The benefits of exercise and physical activity are widely known and have led researchers to become interested in the possibilities it offers as a therapeutic resource of rehabilitation. It has its own characteristics and multiple physiological benefits, according to the “dosage” used, which is why it attracts even more to differentiate its effects in diseases such as PD (18). At the brain level, exercise increases synaptic strength and influences neurotransmission, thus enhancing the functional circuits involved in PD. Furthermore, exercise is a fundamental element of motor learning, influencing, and modifying motor deficit parameters in neurodegenerative diseases (29). The exercise is a fundamental element of motor learning, influencing and modifying motor deficit parameters in neurodegenerative diseases. In PD, the exercise increases synaptic strength and influences neurotransmission, thus enhancing the functional circuits involved in PD (29, 33).

Engagement of PD patients in different types of physical exercise, such as walking on a treadmill, is reported to reduce gait disturbances, to improve executive functions and motor learning (15, 30). Still, gait improvements seem to be specific to the type of motor activity practiced during exercise, whereas improvements in cognitive inhibition are not. This suggests indirect action mechanisms such as enhancement of cardiovascular capacity to. These results are also relevant for the development of targeted aerobic exercise training interventions to improve functional autonomy in PD patients (15). In this sense, regarding exercise dosage, there are various exercise guidelines that generally have a training duration of 4 to 48 weeks and a total of 4 to 96 h, which vary according to the therapeutic objectives and the selected training modality (21). The exercise is usually performed under moderate to high intensity for a long term (34). Mak et al. (19) point out that a training period of at least 6 months is effective to achieve a clinically significant improvement in UPDRS-III scores as well as improvements in gait and walking ability. Here, an intervention time of 12 consecutive weeks is proposed as it is a schedule feasible to be implemented as seasonal “trimestral” (alone or repetitive in an annual schema) schedules in most elderly social center settings (i.e., Winter PasoDoble Program).

As specific resources, improving gait parameters, workouts with differentiation of surfaces, weight support, among others, have also been described (21). These have been combined with signaling strategies, which provide external signals to facilitate the initiation and/or continuation of movement, so patients are instructed to pay attention to signals and to step on the line or markers, or to go to the rhythm of an auditory or somatosensory signal (20, 35). Some examples include visual cues (lines or markers on the floor and on the treadmill), rhythmic auditory cues, metronome rhythms, or music at a preset frequency, somatosensory cues–tactile sensation given to a part of the body as in Tai Chi (a repetitive bodyweight martial art that shifts from one foot to the other, taking a step and turning in different directions) (33, 36, 37).

In this context, Pasodoble is a type of ballroom dance and high groove music. Dance has been incorporated as a rehabilitation strategy since it has sequenced movements performed with music (31, 36). The treatment of neurological diseases is complex due to emotional factors, so dance/movement therapy is of potential benefit in such circumstances. Dance therapy for the rehabilitation of PD (dance/movement) would be more effective than exercise in the outpatient treatment of patients (25, 38) (Hackney and Earhart, 2010). An example is Tango, which improves aerobic capacity, coordination, and balance (26). The benefits observed in dance as therapy can be explained through several mechanisms such as external signals, which can be derived from the music or the couple, as well as the specific movements incorporated in the form of the dance (39, 40). Music provides auditory cues to the premotor cortex through the cerebellum and to the supplemental motor area via the thalamus, allowing the use of these auditory cues to impact individual's gait (35). Some studies indicate that speed, start of gait and cadence are favored with this type of therapy in PD (41, 42). The rhythm of the music and the dance partner can facilitate the coordination of the steps, increase balance and postural stability (43). In turn, signals such as a change in weight and direction of movement given by the dance partner can help initiate the movement and increase or maintain the length and cadence of the steps (Hackney and Earhart, 2010). The dance provides specific patterns of guided movement, as well as proprioceptive and tactile signals in interaction with the auditory signals that the music gives off, facilitating the integration of key elements for rehabilitation in this disease. The current evidence indicates dance, as a complementary therapy, can contribute to the improvement of various symptoms of the patient, both motor, cognitive, and emotional (26, 44, 45).

Dance is a pleasurable and captivating activity that involves motor, cognitive, visuospatial, social, and emotional engagement (39). It is contributing to the configuration of the body schema, the perceptual organization, favoring coordination. Increase body awareness and movement awareness through musical rhythms and melodies (35). It requires spatial integration, rhythm, synchronization with external stimuli, balance, and coordination of the whole body, which makes it a highly useful therapeutic resource for the rehabilitation of PD (25, 35). Furthermore, the dance and movement has a psychotherapeutic, which aims to integrate the individual physically and emotionally, increase the level of personal and body perception, which allows for wide movements and encourages the individual to express himself authentically through the integration of the unconscious (40). The dance rhythmic stimulation may be used to improve gait automation since it helps cognition in the emotional domain, requiring reinforcement in the connections of the frontal cortex and the amygdala (39, 46). In addition, the dance allows participants to establish positive emotional aspects and valence that they experience and store, facilitating knowledge about postures and movements in the most optimal way (47, 48).

Previous studies of dance therapy (Tango, Ballroom, Valls) have shown beneficial results on some gait parameters, as well as an increase in the perception of time, modifying the altered elements for displacement. In turn, improvements in postural balance and motor adjustments for general stability have also been included (46). Dancing in PD promotes greater dynamic balance, increased walking speed, longer step length, positive effects that can be sustained over time (49–51).

Pasodoble is a ballroom where both the person with PD and his/her caregiver (spouse, most of the times) can be involved, although the partner can be another subject or the therapist, too. In this aging population, the role of caregivers for the well-being of the elderly, especially of those affected by neurodegenerative disorders, is crucial (52). In most cases, the spouse or a family member becomes the informal caregiver, holding a demanding full-time responsibility that will worse with the progress of disease with the consequent burden of care (53). The disease severity and duration are determinant factors for caregiver burden, with sleep problems, autonomic dysfunction and psychiatric symptoms amplifying the burden of care in PD (53, 54). Thus, family caregiver burnout in PD is been considered primarily dependent on patient's NMS, requires considerable time investment and can trigger depression in the spouse /family member (53). For the good quality of caregiving and also the quality of life of the physically, emotionally and psychosocially exhausted informal caregiver, early and continued collaboration and support is essential (55). Caregiver unmet needs trigger the requirement of professional care at home or the early institutionalization of the beloved person with PD and the beginning of a new period for family members that may experience anticipatory grief (53). More importantly, increasing number of studies aware of the need to recommend protective therapies for caregivers already in those caring early stages of PD (56). Thus, there's a clear need to provide family-centered care as it is done in oncology (57). As mentioned before, guidelines for family caregiving intervention in PD point at non-pharmacological strategies that include physical-related activities known to improve functional mobility and balance in patients while at the neuronal level exert brain plasticity and neuroprotective effects (17, 58). A comparison of dance interventions in people with PD and older adults points out substantial and wide-ranging benefits but also aware that, as compared to what is known in older adults, more efforts are needed to investigate the growing literature for dance in PD (59).

Dance as part of lifestyle is important, for both patients and caregivers. Prevalence of physical activity in some countries (i.e., the United States) has become a behavioral risk factor (60). The studies aware that an important number of people over age 65 do not meet physical activity recommendations and this situation is worsened in PD (61). In such a case, promotion of social activities that involve physical activities and are part of culture and tradition, like dancing during weekends in elderly social centers or in public areas during festivities, can be more easily implemented. In this sense, dance is presented as a promising intervention because it requires the integration of multiple sensorial information, namely auditory, vestibular, visual, and somatosensory. Besides, any dance style can induce functional adaptations in older adults, especially related to balance as it involves the fine-grained motor control of the whole body (62). Behavioral studies have already provided evidence of better performance in balance and memory tasks in elderly dancers (63–65). The psychotherapeutic use of movement, which aims to integrate the individual physically and emotionally, increase the level of personal and body perception, which allows for wide movements and encourages the individual to express himself authentically through the integration of the unconscious (40). Dance contributes to the configuration of the body schema, the perceptual organization, favoring coordination. Increase body awareness and movement awareness through musical rhythms and melodies. It requires spatial integration, rhythm, synchronization with external stimuli, balance and coordination of the whole body, which makes it a highly useful therapeutic resource for the rehabilitation of PD (25, 35). A recent compilation of research work on sound, music, and movement in PD provided new scientific evidences and arguments highlighting the benefits of therapies using visually, acoustically, and somatosensorially enriched environments (66) but most importantly how the intact ability of PD's patients to pick up and use external sensory information to initiate and time movement would encourage them to actively seek out programs that use sound, music, and movement so that they can lead more active and fulfilling lives (46).

## Conclusions

In summary, founded on evidence-based therapeutic benefits of dance/music therapy we present “PasoDoble,” derived from the widely known Latin ballroom dance traditionally danced in family celebrations and festivities, as a dance/music intervention suitable for family-centered Parkinson's Disease care. As a regular dancing at home, satisfactory “PasoDoble” dance practice can improve and preserve function and mood in the elderly, while in community settings will also promote and improve socialization. The same singular features can be beneficial through rhythmic auditory stimulation and signing for patients with PD since a gait training plan is performed with rhythmic and groove sound stimulation that marks each of the steps in company with the guide of the dance partner, usually the spouse (and family caregiver). The dance partner can help the mastery of balance and displacement by contributing to the control of dynamic balance, controlling external disturbances. We provide “PasoDoble” the methodological proposal and the key features of our protocol, an intervention the method which is structured to improve gait and balance; facilitate movement, reaching and grasping; muscle power and joint mobility; reduce of risk of falls, and increase of aerobic capacity. “PasoDoble” can address each of the four key areas in people with PD on which the benefits of physical exercise in PD are based: to improve gait, cognitive strategies to improve transfers, exercises to improve balance, and exercises to increase joint range and muscle strength to improve physical capacity. Depending on the intensity of the requested exercise, “PasoDoble” can also contribute to improving physical capacity and all its components. Besides, “Pasodoble” is also a music *per se*, with familiarity and groove that compels to move which can serve as an external reference to facilitate specific movements. We consider that this easy-to-implement into patient care and free-living environments (elderly social centers, home) rehabilitation program can promote positive emotions and self-esteem, with added general improvement of social attachment and recognition, thus improving the quality of life of patient-caregiver as a patient and family caregiving intervention that can be tailored to the individuals. The comparison with other ballroom dance and/or rehabilitation circuits and with regards to different stages of disease, will contribute to fully describe its benefits, but also limitations, in different rehabilitation programs and settings.

## Data Availability Statement

The original contributions presented in the study are included in the article/supplementary material, further inquiries can be directed to the corresponding author/s.

## Author Contributions

LG-L: concept. LG-L and LC-M: development of the concept and review, protocol of intervention and graphical abstract, and review and approving final version of manuscript.

## Conflict of Interest

The authors declare that the research was conducted in the absence of any commercial or financial relationships that could be construed as a potential conflict of interest.
